# Comparing professional communities: Opioid prescriber networks and Public Health Preparedness Districts

**DOI:** 10.1186/s12954-023-00840-8

**Published:** 2023-09-01

**Authors:** Patrick Kaminski, Brea L. Perry, Harold D. Green

**Affiliations:** 1 Department of Sociology, Indiana University, Bloomington, IN USA; 2 Luddy School of Informatics, Computing, and Engineering, Indiana University, Bloomington, IN USA; 3 Indiana University School of Public Health, Indiana University, Bloomington, IN USA

**Keywords:** Public health, Social networks, Interventions, Opioids

## Abstract

Problem opioid use and opioid-related drug overdoses remain a major public health concern despite attempts to reduce and monitor opioid prescriptions and increase access to office-based opioid treatment. Current provider-focused interventions are implemented at the federal, state, regional, and local levels but have not slowed the epidemic. Certain targeted interventions aimed at opioid prescribers rely on populations defined along geographic, political, or administrative boundaries; however, those boundaries may not align well with actual provider–patient communities or with the geographic distribution of high-risk opioid use. Instead of relying exclusively on commonly used geographic and administrative boundaries, we suggest augmenting existing strategies with a social network-based approach to identify communities (or clusters) of providers that prescribe to the same set of patients as another mechanism for targeting certain interventions. To test this approach, we analyze 1 year of prescription data from a commercially insured population in the state of Indiana. The composition of inferred clusters is compared to Indiana’s Public Health Preparedness Districts (PHPDs). We find that in some cases the correspondence between provider networks and PHPDs is very high, while in other cases the overlap is low. This has implications for whether an intervention is reaching its intended provider targets efficiently and effectively. Assessing the best intervention targeting strategy for a particular outcome could facilitate more effective interventions to tackle the ongoing opioid use epidemic.

**Supplementary Information:**

The online version contains supplementary material available at 10.1186/s12954-023-00840-8.

## Introduction

During the last two decades, the United States has experienced an escalating drug overdose epidemic of unprecedented scale [39, 48]. Between 1999 and 2020, the number of drug overdose deaths has increased by more than four times from 6.1 deaths per 100,000 people to 28.3 deaths per 100,000 people in 2020 [6]. In over 70% of the recorded overdose deaths, opioids were involved. As a consequence of the increased mortality induced by this opioid-use epidemic, life expectancy in the US has declined over the past several years, with the decline especially concentrated among people in their midlife [54]. Millions of families have been significantly affected by addiction and death [5], and the economic impact of the opioid epidemic in the US is estimated to be in the tens of billions of dollars [38].

In recent years, we have seen a strong rise in mortality caused by illicit opioids like heroin or fentanyl, and much of the current attention is focused on these drugs. However, prescription opioids are still a major contributor to the initiation of non-medical use, opioid use disorder, and overdose deaths [27, 44]. Curbing the opioid epidemic remains a crucial issue that needs to be tackled, and it remains relevant to examine the role providers play regarding access to the prescription opioid supply and to pharmacological treatments for opioid use disorder [7, 8, 17].

Public health interventions focusing on prescribers have had mixed results. Access to a Prescription Drug Monitoring Program (PDMP) has been associated with a decrease in opioid prescriptions but not overdoses [37], and overall, research on the effectiveness of PDMPs in reducing adverse outcomes is inconclusive [43]. A common strategy for implementing interventions (e.g., recent lockdowns or vaccination campaigns) or public health monitoring projects like PDMPs is to introduce a change at the state or community level guided by geographic or political boundaries. However, in the case of opioids, consumption patterns vary largely across different geographic and political areas [41] and localized “hotspots” are common. Recognizing the need to address public health crises on a local level, some states have established a variety of public health emergency preparedness districts as a means to focus their activities better. States group counties into administrative districts that become the basis for addressing regional public health issues.

This study focuses on the state of Indiana which has sorted its 92 counties into ten Public Health Preparedness Districts (PHPDs). PHPDs originated in the early 2000s in response to the 9/11 attacks. At the time, legislators were concerned about potential threats to public health via bio terrorism attacks carried out through pathogens such as Anthrax. Supported by federal funding, states like Indiana have implemented Public Health Preparedness Districts or similar geographically bounded target areas to react nimbly to public health hazards on a more local level. Size, geographic area, access to care, healthcare facilities and existing relationships between Local Health Departments were taken into account for determining the district boundaries.^1^ In theory, local opioid misuse hotspots should be detectable and PHPDs could be used to target interventions and address emerging overdose clusters or related problems.

An complementary data-driven approach for defining public health constituencies could be to use secondary analysis of medical claims data to link providers (here, opioid prescribers) into networks and then into regional communities based on their inferred professional behaviors. Previous research has shown that information diffuses through physician networks [9, 10, 28, 52]. There is also evidence that physician network structure can be leveraged to improve healthcare quality. A study of patient sharing networks [2] found that hospital networks that have more primary care physicians in central positions reported fewer medical specialist visits and lower spending. A study by Cunningham et al. [12] found that cohesive and collaborative healthcare networks facilitate the coordination of care and can lead to improved care. However, research has also identified network characteristics that are negatively associated with healthcare quality, namely the formation of tightly knit groups of providers (cliques), a tendency to form relationships based on professional affiliation or the same gender, and excessive dependence on central actors in the network. This literature suggests that taking professional network structure into account could increase the impact of public health interventions by leveraging the manifold social connections between providers [50].

Physician networks based on shared patients serve as a good proxy for physician communication networks and can be strongly predictive of the diffusion of medical practices [3, 36, 42]. Physicians that share many patients are frequently professional colleagues who consult with each other for advice about new treatments, difficult cases, etc. This has consequences for patient outcomes, as research has demonstrated the important role of social influence and network clustering to the general intensity of treatment and to specific conditions such as treatment for prostate cancer [2, 42]. Physician networks can be mapped based on aggregated medical claims data in ways that protect patient identities but enable detailed insights into the numbers and kinds of patients shared by physicians. Several studies have validated that networks derived from aggregated medical claims data can reproduce and confirm patterns of professional communication that correspond to the results obtained from asking physicians directly about their patterns of communication through a survey [3. 27, 28].

Health insurance claims are the most appropriate data source for this study. Claims provide a very large volume of important and unique information that is impossible to observe using surveys and other methods. Networks derived from aggregated medical claims can comprise nearly complete samples of physicians and their interactions with in-network patients. In contrast, physician surveys are typically limited by self-reporting errors and high non-response rates. Survey non-response is particularly problematic in our study, given that physicians who may be unaware of Office Based Opioid Treatment (OBOT) options or negative views toward it may be less likely to respond to a survey [2, 3, 35, 36, 42].

This study employs network analyses based on claims data to identify professional ‘communities’ of opioid prescribers based on networks built from shared patients. We then compare those communities to groups whose membership is defined by Indiana Public Health Preparedness Districts. First, we determine the degree of overlap between the PHPD and professional community memberships to determine whether empirically generated networks that replicate professional communication patterns correspond well to these exogenously defined administrative districts. Then, turning our attention to non-overlap, we explore whether interventions that operate through the PHPDs might deliver messages to unnecessary recipients while missing key targets. Finally, we compare PHPDs and professional communities with respect to effectiveness at ‘concentrating’ providers linked to key opioid related outcomes: average number of patients with an Opioid Use Disorder (OUD) diagnosis, average daily Morphine Milligram Equivalent (MME) prescribed per provider, average number of patients with an Overdose Diagnosis (OD), average number of Office Based Opioid Treatment patients per provider, and average number of high-risk patients per provider. These analyses will help us determine the relative merits of each of these two methods for grouping and communicating with opioid prescribers. It will also give us insights with respect to strategic health communications related to opioid policies that are aimed at reducing opioid prescriptions. Improved health communications may also drive adoption of OBOT or positive changes in opioid prescribing practices among medical providers.

## Study data and methods

This analysis draws on medical claims data from the Optum Clinformatics Data Mart Database for the period 2017 Q4 to 2018 Q3. The Optum database is a large deidentified database derived from a large claims data warehouse. We removed providers from states other than Indiana.

### Analytic strategy

The analytic strategy we implement is in response to the three primary goals of our study:

1. Recover the community structure of the Indiana provider opioid co-prescription network through patient-sharing ties.

To this end, we constructed a patient-sharing network among providers across Indiana that prescribe opioids. We use the Leiden community detection Algorithm [47] to recover provider communities in which individuals are more densely connected to each other than to members of other communities. These analyses allow us to determine professional communities empirically that are not bound by geographic or administrative constraints but instead are based on professional behavior (prescription patterns).

2. Compare community membership to provider membership in PHPDs and measure the correspondence and mismatch.

To determine whether PHPD interventions might also be gaining the benefit of patient-sharing networks, we compare the overlap between PHPD and community membership. Assessing the match or mismatch between co-prescription network communities and PHPDs can provide crucial insights about whether delivery of interventions and strategic communications via geographically determined PHPDs could be augmented with a network-based delivery mechanism to better combat the ongoing opioid epidemic in Indiana.

3. Assess which means of targeting (PHPDs or professional communities) may better reach key providers in the context of the opioid epidemic and which divisions may ‘concentrate’ opioid outcomes more effectively.

It is unknown whether opioid-related outcomes of interest are more concentrated within PHPDs or provider communities. Lastly, we explore the possible over- and under-reach of interventions that focus solely on administrative divisions by calculating the number of providers that might be missed by relying only on PHPD boundaries for network interventions rather than adopting a strategy that combines PHPD-focused interventions with community-focused interventions.

### Network construction

Because the focus of this study is opioid-related provider behaviors and outcomes we chose to focus on opioid co-prescription networks. We removed all providers that did not prescribe any type of opioid to any patient. While providers share patients for many different reasons, the focus of this study lies in behaviors and outcomes related to opioid prescriptions. Thus, with respect to network ties, only opioid prescriptions are considered valid for constructing the patient-sharing network. We excluded other prescriptions or non-opioid-related patient-sharing for these analyses. For example, if two providers share two patients—A and B—through co-prescription but only prescribe opioids to patient A, then only the tie to patient A is considered for network construction. Single provider–patient pairs with no other connections are not included because they are not part of any larger patient-sharing network based on our data.

We then construct a two-mode network in which providers and patients are linked through opioid prescriptions. In the next step, we project a one-mode network in which ties are created between providers if they share a patient through opioid prescriptions. For example, if providers A and B prescribe opioids to patient Z a tie will be constructed between providers A and B in the one-mode projection. The final step of network construction is the extraction of the largest connected component. Since we are interested in providers that are part of a network community, isolated providers and providers that are not connected to the largest connected component (the largest component in the network in which every node can be reached through some path) are removed from the network.

### Community detection

Community detection is the standard approach to find groups of nodes within a network that have a higher probability of being connected to each other than to other nodes in a network. There are a wide variety of approaches with different underlying assumptions used to cluster networks [19], and there is no single algorithm that performs best across different types of network [40]. However, systematic comparison of different community detection approaches has shown that the Louvain algorithm performs well across a wide range of networks [55]. For this analysis, we chose to use the Leiden algorithm [30, 47], an improved version of the older and highly cited Louvain algorithm [4].

### PHPD assignment

**Fig. 1:**
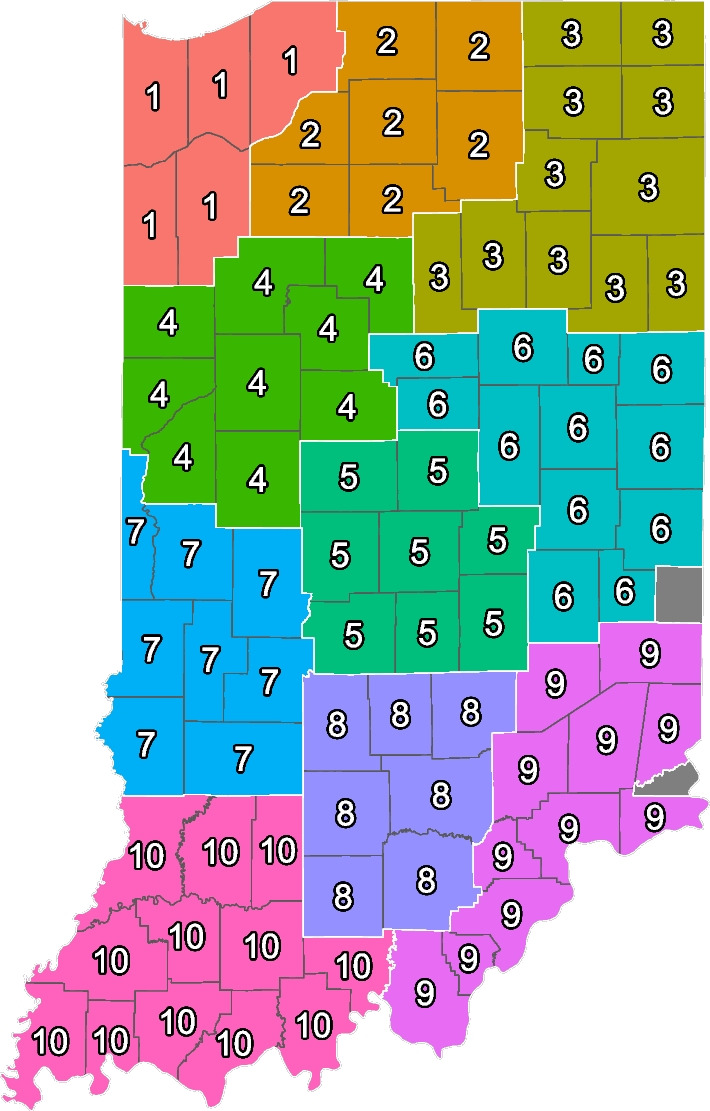
Public Health Preparedness Districts. PHPD membership for each county. Number within the county is the number of the PHPD the county is a member of. Colors correspond to PHPD numbers. Darker lines represent county borders. White lines correspond to PHPD boundaries. 90 out 92 counties in which we were able to infer provider location are included

The database used in this analysis provides detailed information on the ZIP5 level code of patients, but only specifies the state of a provider. However, to assign every provider a membership in a PHPD we need the county location of a provider. To approximate provider location, we use density-based spatial clustering of applications with noise (DBSCAN) [15]. DBSCAN identifies clusters with many nearby neighbors to infer the approximate location of an entity. This is the most commonly used algorithm to infer geographic location; thus, we use it to infer the location of providers based on the location of their patients. Every provider is assigned the most likely county in which they practice. We could identify providers in 90 out of 92 counties in Indiana in the data set; for two of our 92 counties, we could not identify any provider based on the geographic composition of their patient population. Figure 1 shows the distribution of PHPDs across the state and the boundaries of every district.

### Clustering similarity

To assess similarity between different groups, we need to use a systematic method of comparison. There are many clustering comparison approaches available, yet they often have biases (such as the inability to compare clusters of varying size) that can lead to dramatically different results as recent research has shown [22]. This is particularly important for clustering in networks because network clusters often embody a heterogeneous structure with different cluster sizes that have to be taken into account. For example, it is reasonable to expect that clusters in a metropolitan area will be larger than clusters in a rural area due to a different number of providers based on geographic location. The clustering similarity method developed by Gates et al. (ibid.) avoids these shortcomings and offers a unified method to compare clusters based on their individual elements.

Instead of focusing on clusters themselves, clustering similarity looks at the relationships that are induced by the clusters. In this case, we are interested in the relationship between provider communities and PHPDs and clustering similarity allows us to assess the differences at the individual provider level. For every provider, a similarity score is calculated [21] that indicates the similarity between the composition of the network community the provider is part of and a provider’s administrative clustering membership. If a provider’s neighbors across both clustering methods are the exact same their similarity score will be 1, if a provider’s neighbors are entirely different their similarity score will be 0. We then aggregate the similarity score as a population average on the county level to see how much PHPD and provider communities overlap.

### Concentration of outcomes

To assess differences in the distribution of relevant outcomes (OUD, OD, Average MME in the provider population, number of high-risk patients^2^), we calculate for each community and PHPD a weighted population average for each outcome and then calculate the Gini coefficient. The Gini coefficient is a commonly used method for assessing inequality within a set of observations [16] and gives us a measure of to what extent outcomes of interest are unequally distributed within different clusters. A Gini coefficient of 1 indicates maximum inequality; a coefficient of 0 indicates maximum equality. This is relevant because better concentration provides key insights for choosing which areas to prioritize to intervene on first or in what order. For example, if the distribution of overdose outcomes is dissimilar between clusterings (e.g., the Gini coefficient for overdoses in clustering A is closer to 1 than in clustering B), we can assume that one clustering better represents the concentration of this outcome than the other clustering and is therefore better suited for a potential intervention.

## Results

1. Recover the community structure of the Indiana provider opioid co-prescription network through patient-sharing ties.

To address our first aim, we recovered the community structure of the Indiana provider opioid co-prescription network. As an important step before analyzing communities, we need to assess how sensitive our community detection results are to different starting conditions. Community detection algorithms depend on random seeds to set the initial conditions for every fit. This initialization can affect results. To test the sensitivity of our community detection methods to using different seeds, we ran the community detection algorithm 15 times with different random seeds. In the next step, we compared the community detection results to each other (e.g., A to B, A to C, etc.). A score of one indicates a perfect match (all providers are in the same community across different fits); a score of zero indicates complete dissimilarity (all providers are in completely different communities). Generally, the community detection results differed slightly based on different initialization conditions. On average, the community detection results were 88% similar (score of 0.88) with a standard deviation of 0.03, a minimum similarity of 80% (score of 0.8), and a maximum similarity of 95% (score of 0.95). Therefore, the results are relatively robust no matter what the initialization conditions are. We detected twelve provider communities in Indiana with an average of 600 providers (median = 452.5) and a standard deviation of 680 providers. Community size ranges from 2,532 for the largest community to 23 for the smallest community. We used these numbers for all subsequent analyses.^3^

2. Compare community membership to provider membership in PHPDs and measure the correspondence and mismatch.

**Fig. 2:**
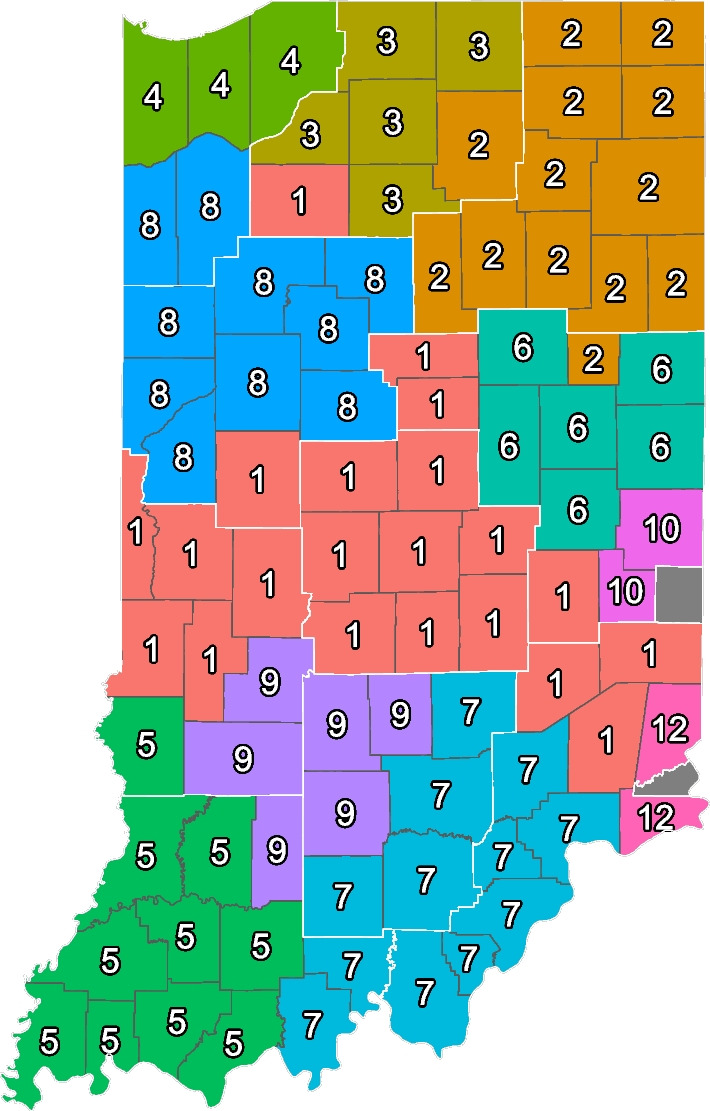
Distribution of Largest Community per Indiana County. Professional community membership for each county. Number within the county is the number of the predominant community in each county. Colors correspond to professional community numbers. Darker lines represent county borders. White lines correspond to PHPD boundaries

Our second aim was to compare community structure to provider PHPD memberships and to measure the overlap or mismatch. Figure 2 summarizes the findings by displaying the largest community within each county. The white borders around the districts are the boundaries of the PHPDs. This map shows that in some areas, majority communities correspond well to existing PHPD district borders, while other districts are split between multiple communities. For example, community 2 in the northeast of Indiana matches well with PHPD 3. However, community 1 sprawls across six different PHPDs with varying levels of correspondence. While this figure is only based on the largest provider communities in a county, our subsequent analyses quantify overlap between PHPDs and professional communities. The first figure shows the distribution of the ten PHPDs in Indiana. The white borders around the districts are the boundaries of the PHPDs.

The second figure shows the distribution of communities across the state. Many counties have multiple communities represented. For simplicity, this map shows the largest representation for every county and the map preserves the boundaries of the PHPDs. The degree to which communities align with PHPD varies significantly. For example, the counties included in PHPD 3 and community 2 look fairly similar. The same can be said for PHPD 5 and community 5. However, there are also cases where the opposite is true. For example, community 1 spans most of Central Indiana and is the major community in counties included in six different PHPDs. In PHPD 5, which contains Indianapolis and its adjacent counties, community 1 is the sole majority community of all counties. It is also dominant for some counties in PHPDs 4,6,7, and 9 but is far from dominant in comparison with PHPD 5. This suggests that in the PHPDs surrounding PHPD 5, correspondence between PHPD and community membership should generally be lower because professional communities are not in line with PHPD boundaries as was the case for PHPD 3.

This measure of correspondence at the county level is useful for visual representations, but it is rather crude. For greater detail, we can analyze individual provider similarities using clustering similarity methods [22] which we will do in the next step.

Overall, PHPDs and professional communities in Indiana have a correspondence of 69% percent (0.69). This means that on average 69% of the providers share the same PHPD and professional community. While this average value is relatively high, it means that about a third of providers belong to a professional community that does not align with their PHPD. Furthermore, one cannot make conclusions about local similarity from this aggregate number. Assessing provider similarity scores at the county level results in a more nuanced picture. Some counties have a very high similarity score of over 90% such as Daviess, Gibson, Knox. Other counties have a very low score such as Sullivan with 2% and Vermillion with 3%. We should note, however, that the ten largest counties by provider population in our sample have scores ranging from 54% (Madison) to 91% (Vanderburgh).

**Fig. 3:**
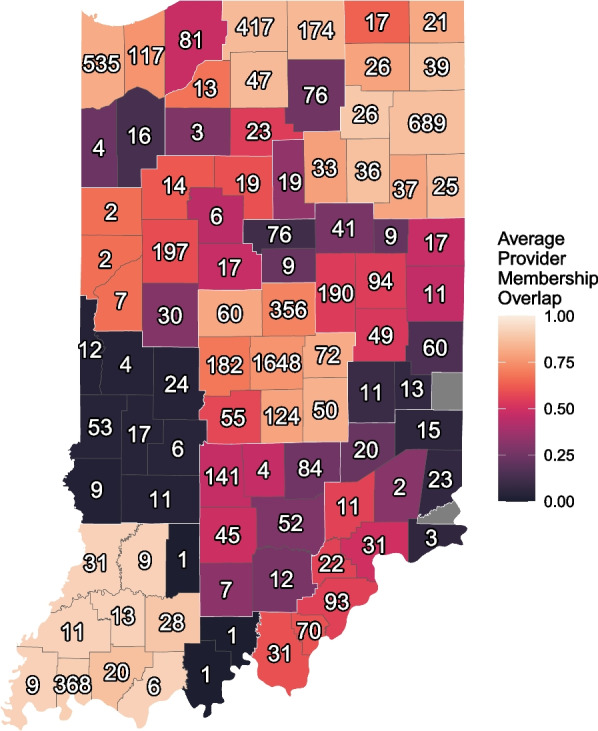
Average Provider Similarity Score for Counties in Indiana. We calculate the average similarity score for every provider and aggregate it at the county level. The thin dark lines represent county borders. The white lines correspond to the PHPD boundaries. Low scores (darker color) indicate that providers in this county tend to be clustered with a different set of providers in each group. A high score (lighter color) indicates that providers in this county tend to be clustered with the same set of providers in each group. The number within the county represents the total number of providers we could identify in the county

Figure 3 shows a more detailed view of provider correspondence. We calculated the average similarity score for all providers in a county. Lighter colors indicate higher similarity; darker colors indicate lower similarity. The number in the center of each county is the total number of providers in each county. For example, looking at PHPD 3 in the northeast shows that most of the counties have very high average similarity scores, indicating that the correspondence between PHPD membership and professional community membership is high. The opposite is true for PHPD 7 in the west of Indiana. Average provider similarity scores are among the lowest for every county and PHPD, indicating that the overlap between professional communities and PHPD districts is very low. Looking at Fig. 2 holds the key to explaining this observation: PHPD 7 is divided across three different communities which explains the low similarity between clusters.

It is also noteworthy that counties with fewer providers seem to have a lower average similarity score than counties with a larger provider population. To test this, we calculated the Pearson correlation coefficient and found a statistically significant (*p* < 0.02) positive correlation of 0.25 between number of providers in a county and level of correspondence between PHPD and professional community membership. This suggests that smaller counties with fewer providers also tend to have lower similarity scores in our sample.^4^

3. Assess which means of targeting (PHPDs or professional communities) may more effectively reach key providers when precision targeting is needed and which divisions may ‘concentrate’ opioid outcomes more effectively.

To assess the potential impact of professional network and administrative district mismatch, we calculated some descriptive statistics focusing on provider concentration in communities and calculating Gini coefficients to compare concentration of important outcomes between professional communities and PHPDs.

Table 1 shows the total number of providers in a PHPD, the community with the largest population in the PHPD, the number of providers in the largest community and the percentage of providers from the PHPD that are also members of the largest community (and, by extension the percentage of mismatched providers in each PHPD). The goal is to explore how many providers could be reached (and might be missed) through a network intervention targeting the main professional community in a PHPD and how many providers may be unnecessarily contacted if we used a PHPD-focused broadcast intervention. On average, a network-based intervention in a PHPD would reach around 79.7% of providers in any particular PHPD. However, the PHPD/community correspondence of the provider population ranges widely from 52.4 to 98.3% across PHPDs. This can lead to substantially different regional outcomes. For example, in PHPD 3, around 94% of providers in the PHPD share the same professional network community and a network intervention targeting the primary community in a PHPD could be quite effective. The opposite is true of PHPD 8. A network-based intervention in this district has a much lower chance of reaching a large proportion of the targeted population because only roughly half of the provider population (52.4%) is connected through the primary professional network.

**Table 1:** Percentage of providers in majority community

PHPD	Number of providers	Majority community	Number of providers in the community	Percentage of providers in largest community
5	2547	1	2193	86.1
3	968	2	914	94.4
2	753	3	667	88.5
1	753	4	649	86.1
10	498	5	490	98.3
6	580	6	354	61.0
9	321	7	247	76.9
4	294	8	244	82.9
8	345	9	181	52.4
7	136	1	96	70.5

It should be noted that in addition to PHPDs we considered several other regional divisions such as Economic Growth Regions (EGR), Bureau of Developmental Disabilities Services (BDDS), districts or Core-based statistical areas (CBSA). Consultation with key informants associated with the Indiana State Department of Health led to the choice of PHPDs as the most relevant regional division to assess. However, the second most promising contender for the comparison was Hospital Referral Regions (HRR) and we compared our PHPD analyses against similar analyses with HRRs. HRRs represent regional health care markets for hospital care [13]. Previous research has used HRRs to define discrete health care populations [32]. Unlike in our data in which every provider can only be member of one community and one county, some counties are members of multiple HRRs. Therefore, in counties with more than one HRR our providers can be members of any of the HRRs in that county. To account for this, we assessed the overlap between HRR membership and PHPD membership in two ways: First, we assigned every provider to one HRR and if a provider had more than one potential HRR membership we randomly sampled from the pool. We repeated this procedure 1000 times, aggregated the scores and calculated the average clustering similarity across the random assignments. On average, the clustering similarity score between PHPDs and HRRs is 62.75% with a standard deviation of 3.16% which is lower than the clustering similarity between professional network communities and PHPDs. Second we ran a multimembership comparison in which providers are members of all potential HRRs. In this analysis, the overlap was 37.33% which is again lower than for the professional network communities. Lastly, we assessed the overlap between professional network community membership and HRR membership. The average overlap for a single assignment is 56.09% with a standard deviation of 2.23% and 36% for the multimembership comparison. Professional network communities and HRRs have a relatively low overlap, indicating that current health care market boundaries are not a good reflection of opioid co-prescription patterns. The highest overlap between all evaluated clusterings is between professional network communities and PHPDs.

Finally, to assess whether PHPDs or professional communities might make better ‘catchment zones’ or targeting divisions, we looked at how well each of the divisions aggregated patients according to key outcomes associated with opioid prescriptions. The outcomes were average daily MME prescription per provider, number of patients with an OUD diagnosis per provider, average number of patients with a non-fatal overdose diagnosis per provider, number of OBOT patients per provider, and number of high-risk patients (a combined risk class defined as individuals with significantly higher average daily MME and greater likelihood of SUD diagnosis) per provider. Accounting for the patient population size of a provider, we calculated weighted averages for these outcomes grouped by PHPD membership and by professional community membership. Next, we calculated the Gini coefficient to estimate the distribution of outcomes between different groupings. The Gini coefficient ranges from 0 (total equality) to 1 (total inequality). Across all outcomes, the Gini coefficient is higher at the professional network community level. The degree of inequality varies, however. For example, the average daily MME coefficients are relatively similar but for other outcomes the differences are more pronounced. These results, summarized in Table 2, show that key outcomes related to opioid prescriptions are consistently more concentrated in professional communities rather than in PHPDs, which has implications for more effective targeting of opioid-related public health campaigns for providers as well as patients.

**Table 2:** Gini coefficient table for key outcomes associated with opioid prescriptions

Gini coefficient	Average daily MME per provider	ORD patients per provider	Average number of overdose diagnoses	MAT patients per provider	Number of high-risk patients per provider
PHPD	0.12	0.38	0.28	0.36	0.35
Community	0.15	0.48	0.38	0.53	0.44

## Discussion

In this study, we compared the twelve professional communities formed by shared patients in our claims data (as described previously) against other health-related administrative districts. After considering several administrative divisions of the state including workforce development zones, economic growth regions, state police regions, disabilities services regions, and Public Health Preparedness Districts, it was determined that the most appropriate comparison for our study was to Indiana’s Public Health Preparedness Districts (PHPDs), which cluster counties into 10 geographic regions. After assigning each provider their respective professional community membership and their PHPD membership, we assessed how well these divisions reflected each other. We did the same at the county level.

To assess the robustness of our findings, we conducted similar analyses for Hospital Referral Regions, a service delivery-based division of the state, but found stronger overlap between PHPDs and co-prescribing networks than between HRRs and co-prescribing networks OR between PHPDs and HRRs. Thus, we focus this discussion on our PHPD analyses.

We found that levels of overlap varied, with some PHPDs and professional communities almost entirely the same in some areas of the state while drastically different in others. We also showed that this mismatch is most extreme in counties with fewer providers.^5^ Across the state, there was about a 20% mismatch, suggesting that if we asked providers to share important messages with their colleagues based on targeting within PHPDs, 1 in 5 doctors would be unlikely to receive the message through their professional networks and if we broadcast to a particular PHPD we would be likely to overdeliver to 20% of the providers, wasting precious resources by informing all doctors in an area where only 80% might need the message. This suggests that in certain circumstances where there is a need for rapid, targeted and efficient response, alternative mechanisms for targeting strategic health communications may be more efficient and cost-effective (e.g., network-based strategies) for delivering certain messages. We created maps to display this information and encourage further discussion among public health policymakers.

To assess why this is important in the context of the Indiana opioid crisis, we also explored the prevalence and distribution of average daily MME prescriptions, OUD, overdose, and Office-based Opioid Treatment (OBOT) prescribing within the PHPD framework and our professional community framework. To compare the two administrative frameworks, we used a statistical index that assesses inequality (i.e., heterogeneity or variation) across regions called the Gini coefficient. This index is often used to assess international wealth inequalities but can be used to assess inequalities across any indicator. Our position was that if the Gini coefficient was higher for one or the other of these ways of dividing the state, then that division was doing a better job at identifying areas of most concentrated need and thus would suggest which of the approaches—PHPDs or professional communities—might be a better way to selectively target communications, reach priority providers, and more effectively use important resources for addressing addictions in Indiana. It is noteworthy that some measures such as average daily MME per provider have relatively low levels of intergroup heterogeneity regardless of the manner by which the providers are grouped, while the other measures have higher levels. This is likely due to federal and state regulations and policies that limit opioid prescribing for all providers in the state. Other outcomes are patient-related which are unconstrained by such restrictions and are largely related to the provider’s own professional activities or their patients’ opioid-related health outcomes.

Regardless of the specific level of intergroup heterogeneity, across all measures calculated, our network-based professional communities reported higher Gini coefficients, indicating greater heterogeneity across the communities (thus better concentration of our indicators within specific communities) and suggesting that targeting providers in those communities (or the counties where the majority of providers in those communities practice) might be more effective and economical than a state-wide or PHPD-based approach in the specific context of the Indiana opioid crisis.

We note, however, that this set of analyses is designed specifically for understanding opioid-related behaviors and thus may not generalize to other sorts of substances such as benzodiazepines or even to illicit opioids. However, we believe that drug-seeking behaviors and regional substance use issues may be similar enough that we could rely on these analyses for related provider-focused campaigns. We are not as confident, however, that these analyses generalize to other provider-focused campaigns that target infectious diseases such as HIV (where a different set of provider communities may emerge) or to broader epidemiological issues that focus on pandemic response where the geographically clustered PHPDs may be more efficient and effective for communication.

This research represents a shift toward a relational conceptualization of professional decision-making around opioid-related provider behavior. The findings are particularly relevant to interventions where diffusion of information or social norms through provider networks are likely to be more effective. We focus here on opioid-related professional behavior, arguing that empirically developed and analytically tested strategies could improve provider responses by increasing providers’ knowledge of and willingness to accommodate policy changes, particularly when they might face cultural and professional barriers, such as the decision to limit opioid prescriptions or to prescribe buprenorphine. As with many professional behaviors that have a cultural and social significance, opioid-related professional behavior may be affected by the attitudes and behaviors of others present in a provider’s network of professional connections. Professional networks shape individual provider attitudes, beliefs, and behavior through three mechanisms: (a) providing exposure to new treatments through social influence; (b) providing information to reduce uncertainty around treatment effectiveness and risk through social learning; and/or (c) affecting the care provider’s beliefs and attitudes through social norms and monitoring mechanisms. We have determined that opioid-related behaviors do cluster in local sub-communities, impacting the identification of appropriate intervention targets for information campaigns and identification of possible provider ‘change agents’ for peer-based interventions based on social learning and social influence processes.

Because we know that OUD behaviors cluster more strongly within professional communities than in PHPDs, stigma-reduction and knowledge-building campaigns targeting professional communities that have low prescribing relative to the prevalence of OUD and heavy opioid prescribing or that may have more stigmatizing attitudes than other communities may be more effective than state-wide campaigns or even PHPD-focused campaigns. Given the importance of social influence on behavior, we might consider contacting the sorts of providers that are most central in these professional networks, who are likely to have the greatest ability to influence the behavior of their peers. Alternatively, higher level prescribers in each professional community might be the best sources of information to encourage changes in the prescribing behavior of their low-prescribing peers. If OBOT prescribing is driven by exposure to specialists or other OBOT providers, then targeted educational outreach using pain specialists or other OBOT providers (i.e., change agents/champions) would be recommended. It may be that encouraging these change agents to recommend that their colleagues increase their level of prescribing will best facilitate the diffusion of the OBOT outcomes. Structural changes in the network (i.e., creating new professional relationships between providers) may also impact OBOT prescribing. It may be that forging a new consultative relationship between a prescriber and a non-prescriber is enough to achieve a change in non-prescriber behavior. Regardless of the strategy chosen, network-based targeting of strategic information campaigns and implementation strategies may lower costs and increase the impact of such campaigns relative to more traditional approaches in certain circumstances [14, 23, 25, 46, 50, 51].

This study demonstrated that the overlap between PHPD boundaries and the community structure of provider networks varies significantly based on geographic location (and also likely on the public health context in question). While the overlap is high in some areas, other areas have low overlap. If the goal is to have effective interventions to target specific groups of providers or patients in the context of a specific public health concern, then in some cases a community-based approach might be more effective. One solution to this problem could be the use of data-driven community-determined divisions based on professional connections such as patient-sharing for strategic communications. In this case, some PHPD districts with high similarity to professional communities would be used in their current form while other districts with low similarity might be reorganized into new ones that more closely align with naturally occurring and empirically determined provider networks. This ‘as-needed’ context-based approach would incur organizational cost and would necessitate changes in recommended procedures at the state level but could lead to highly efficient interventions that could rely on existing professional provider networks specific to the particular public health challenge. Indeed, in the most extreme case, this community detection and district formation process could be undertaken each time a new public health concern arose using data specific to the public health challenge, though this is likely unnecessary.

While we cannot make claims about what the exact best approach for data-driven, context-based regional targeting of interventions is, other disciplines have proposed solutions similar to our proposed computation approach. For example, gerrymandering of congressional districts is a widespread problem in the US. In response to this, Computational Redistricting [33, 34] is a method that takes relevant factors (e.g., population composition, majority-minority distribution, district packing and cracking) into account and creates new districts optimized for desired outcomes such as competitiveness. In the case of opioid prescriptions, relevant factors might be, for example, professional communities among providers, geographic distance, and epidemiological characteristics that could be used to create targeting regions that are more efficient at broadcasting interventions while maintaining geographic coherence. It should be noted that targeting regions for other public health interventions (e.g., HIV prevention) might look different due to different professional network ties.

We believe this study makes three significant contributions to improve population health: First, this research provides empirical evidence regarding targeting, relative reach and possible performance of strategic communication strategies targeting opioid and opioid therapy prescribers. Changes in policies related to opioid prescribing and access to office based opioid therapy (OBOT) have produced active debates about the impact of expanding the availability of opioids and OBOT not just among providers but among harm reduction and OUD prevention advocates [20, 24, 29, 31]. Strong opinions exist within the medical and advocacy communities, underscoring the need for new empirical and theoretical approaches to guide policy change and develop effective policies. Second, the analyses in this study facilitate the targeting of strategic information campaigns and implementation strategies and thus may lower costs and increase the impact of such campaigns when we consider peer-based strategies relative to a more general and widely implemented ‘broadcast’ type intervention. Third, while these strategies have been explored in the context of opioid related policies, these methods can easily be generalized for use in the context of any new SUD treatment modality that would benefit from targeted implementation strategies [1], particularly those for which social learning, social influence or other network-based phenomena are expected to operate. Indeed, any novel medical innovation that faces barriers to implementation based on factors beyond provider knowledge may benefit from the findings of our study. Thus, this study has broad relevance for addressing not just substance use in the United States but public health in general. However, as the nation’s focus shifts to other substances driving increased morbidity and mortality (e.g., methamphetamine) [53], and promising new treatments for methamphetamine use disorder are developed [49], the fundamental social processes we explore and the methods we employ in these analyses can be directly transferred beyond the context of the opioid epidemic.

This research introduces some key innovations to the study of opioid use and opioid use disorder. It is among the first to apply social network analysis to the study of professional communities in the context of opioid and OBOT prescribing and possibly the first to compare those communities against existing administrative divisions. Previous studies have explored provider/patient networks; however, the majority of those studies focus on patient networks and patient outcomes or the patient/provider relationship in the treatment cascade rather than focusing on provider/provider networks and provider behavior (i.e., the decision to prescribe opioids or provide OBOT) [11, 45]. This study advances science by answering questions regarding the feasibility of network-based targeting approaches in the context of opioid use, moving beyond studies of provider- or organizational-level factors related to opioid and OBOT prescribing to examine the social and professional-level factors that impact provider behavior. These approaches can inform the possibility of new network- and peer-based opinion-leader interventions. Peer-based interventions may more effectively address critical drivers of unmet treatment need—lack of information about opioid related policies and treatments—than standard drug detailing behaviors, mass media campaigns or continuing education.

This study has several limitations. While DBSCAN is the standard method to infer the approximate location based on the surrounding clusters, it can lead to false classifications for example if a provider serves a high proportion of patients from outside their own county. This may be the case for specialist providers. In some areas, certain provider specialties are scarce and likely draw a diverse population from multiple counties. However, this is likely only to impact specialists that serve a smaller number of patients than their colleagues. Another limitation is that some counties are sparsely populated, and therefore, it is harder to detect the exact location of providers. In this particular case, some counties only have one identified provider; therefore, one needs to proceed with caution interpreting the results for those counties. However, both low and high similarity are observed across counties with high provider counts which indicates that the results overall are not driven by small n observations.

Our analyses in their current form are only applicable to opioid prescribing behaviors because we did not include any other substances in this study. While other prescription drugs contribute to overdose deaths, opioids remain the primary cause of overdose deaths and thus are the focus of this study. We cannot make claims with regard to the patterns that might be observed for other controlled substances but we expect we might find similar patterns.

We can also not make any claims with regard to diffusion efficacy. Previous research has shown that network interventions can be highly effective [18, 26], it is not clear if that is true for opioid-related interventions. Simulating the impact of these strategies and gathering information on providers before implementation may further reduce implementation costs and increase cost-effectiveness of OBOT-related campaigns. Future simulation studies could address this gap. Future research will explore simulations that compare broadcast-type interventions and staged regional interventions (PHPD and professional community) against network-based popular opinion leader/mentor and change agent-type approaches to assess how they impact outcomes of interest. These network-based interventions move beyond standard continuing medical education approaches and broadcast-type messaging to better target the counties and professional communities most affected by OUD and least likely to prescribe OBOT.

### Supplementary Information

**Additional file 1.** This table shows the distribution of providers per network community. Every provider is a member of one community.

## Data Availability

The data that support the findings of this study are available from Optum Clinformatics Data Mart Database but restrictions apply to the availability of these data, which were used under license for the current study, and so are not publicly available.

## Abbreviations

DBSCAN: Density-based spatial clustering of applications with noise
MAT: Medication-assisted treatment
MME: Morphine Milligram Equivalent
OBOT: Office Based Opioid Treatment
OD: Overdose
ORD: Opioid-related disorder
PHPD: Public Health Preparedness District

## Footnotes

1 This is based on public information and personal correspondence with public health officials in the state of Indiana Department of Health. To our knowledge, no other documentation is available.

2 To identify the risk level of patients, we generated a latent variable through Gaussian finite mixture model-based clustering of three observed variables: the average daily MME prescribed opioid drug use, opioid use disorder and overdose diagnoses.

3 A table with the full distribution of providers across communities can be found in the Additional file 1.

4 It should be noted that for eleven of our counties we could only identify four or less providers per county. However, given that the correlation is moderate and there is significant variation even for counties with larger provider populations there is no indication that these counties drive our overall results.

5 We also tested if a) there is a relationship between average similarity score in a county and if it is deemed a Health Care Professional Shortage Area (HSPA) and b) if non-metro areas are more likely to be a designated HSPA. In both cases, correlations were insignificant suggesting that there is no relationship between similarity scores at the county level and HSPA designation or HSPA designation and being a non-metro area.
