# Supplementary material for: Comparing professional communities: Opioid prescriber networks and Public Health Preparedness Districts
Source: Harm Reduct J. 2023 Sep 1;20:120. doi: 10.1186/s12954-023-00840-8 (PMC10474636; doi:10.1186/s12954-023-00840-8)
Supplement: Supplementary file 1 — Additional file 1. This table shows the distribution of providers per network community. Every provider is a member of one community. [file 12954_2023_840_MOESM1_ESM.pdf]

Appendix

Table 1. Distribution of providers across communities

Community	Number of Providers
1	2532
2	1023
3	764
4	662
5	533
6	503
7	402
8	367
9	277
10	65
11	44
12	23
